# An early start at a professional soccer academy is no prerequisite for world cup soccer participation

**DOI:** 10.3389/fspor.2023.1283003

**Published:** 2023-11-23

**Authors:** Sebastiaan Willem-Jan Platvoet, Germen van Heuveln, Jos van Dijk, Tom Stevens, Mark de Niet

**Affiliations:** ^1^ Research Group Talent Identification & Talent Development in Sport, School of Sport & Exercise Studies, HAN University of Applied Sciences, Nijmegen, Netherlands; ^2^ School of Sport Studies, Fontys University of Applied Sciences, Eindhoven, Netherlands; ^3^ Invictus Sport, De Meern, Netherlands

**Keywords:** talent identification, talent selection, football, qatar22, youth career, pathways, FIFA, talent development

## Abstract

**Introduction:**

829 players from 32 nations on five continents participated in the 2022 men's World Cup tournament in Qatar. Not much is known about the youth careers of World Cup players from all over the world, especially about the age at which they began playing youth soccer in a professional academy. This study aimed to provide insights in the age national team players participating in World Cup Qatar 2022 started playing for a professional soccer academy and whether their starting age relates to continent and their current playing position (i.e., goalkeepers, defenders, midfielders, and forwards).

**Method:**

Systematic online desk research was conducted to determine the age at which World Cup players started playing for professional youth soccer organizations. The median and interquartile ranges were expressed for the starting age in professional youth soccer organizations and the current age at the World Cup. The variables were compared with playing position, the continent of the player's World Cup nation, and the continent on which the player was raised.

**Results:**

The results reveal that World Cup Qatar 2022 players started playing for professional soccer academies at a median age of 13.2 years (range: 4.2–22.6). In Europe, players started playing for professional youth soccer organizations earlier than players on other continents [*χ*^2^ (4) = 142.0, *p* < 0.001]. We also found a younger starting age in forwards than goalkeepers (*p* < 0.05).

**Discussion:**

In most established soccer nations in Europe and South-America, World Cup players started playing for professional soccer academies before the age of 12. However, a significant number of players started later, especially players on other continents, which reveals the different pathways youth players can follow to the elites.

## Introduction

Soccer is by far the most popular sport in the world and is played by many millions of athletes worldwide. Many young people dream of playing for their national team and competing at the Fédération Internationale de Football Association (FIFA) World Cup. At the 2022 men's World Cup tournament in Qatar, 32 of the 210 FIFA member nations each sent 25 or 26 players who competed to become world champion. Before they compete at an elite level, athletes practice their skills from young ages and often for more than 10 years. Although recent studies (e.g., Güllich, Barth (1–3) have questioned early selection and specialization, little is known about the pathways and ages at which World Cup players started playing soccer for professional soccer academies. A better understanding of when these 829 World Cup players first joined professional soccer academies and how that relates to their continent, nation, and playing position might help to clarify current talent identification methods and development processes worldwide.

For a nation to compete and be successful at an international level, high-standard talent identification and talent development programs are assumed to be prerequisites (4, 5). A key factor in the development of elite athletes is the activities and pathways in which they engage during their youth career (6). Much has been written about the early specialization pathway and the early diversification pathway, which Ford and Williams (7) suggest are opposite ends of a continuum. However, most elite athletes are probably not at one end of that continuum. For example, Hornig et al. (8) showed that senior national team players in Germany follow different career paths than senior amateur players with similar youth paths (8). Both started playing unorganized soccer at age 4 and organized soccer on average from age 5. However, the senior national team players were more likely to continue playing unorganized soccer more until age 10.

Despite evidence that selection at young ages may not be a good predictor for future success (1, 8, 9), many soccer organizations—especially in established soccer nations—invest considerable resources in identifying promising youngsters in the sampling years, i.e., between ages 6 and 12 (10). Selection at young ages stimulates early specialization (i.e., a strong focus on sport-specific performance development *via* deliberate practice), the wisdom of which has been questioned and is still under debate in the literature (6, 11, 12). However, players who are selected early do generally receive better opportunities (e.g., higher quality coaches, equipment, teammates and opponents) to develop themselves (7). For example, greater engagement in soccer-specific play activity in the sampling years has a positive influence on the decision-making ability of elite soccer players (6). These contradictions in the literature suggest that much is still unknown about identification, selection, and development at young ages and more research into young players is warranted.

In addition, much is unknown about talent identification and talent development in emerging soccer nations since athletes from Asia and Africa are underrepresented in talent identification studies (1, 13). This limits our understanding of current talent identification and talent development processes worldwide, especially as nations have different talent development systems (14).

In Europe and South America, professional soccer clubs play an important role in identifying and developing players in the first phase of their career. There are also national-level under-15 teams in those countries. However, those teams select no more than 18 players a year, and only a few of the players on youth national teams make it to the senior national team (3). Therefore, the professional soccer academies of elite clubs in Europe and South America remain the most important organizations in supporting and developing future elite soccer players, including those who will play on national teams.

Funding by the government or private organizations plays a dominant role in talent identification and talent development processes in Asia, Africa, North America and Central America. In a school-sport system, resources are concentrated on developing sport talent either within the curriculum and/or through extra-curricular activities (15). These school-sport organizations begin usually at high school level, so athletes are not selected before the age of 12. It should be acknowledged that in Asia, Africa, North America and Central America soccer is most often not the most practiced and popular sport in contrary to Europe and South America. Whereas in most countries in these two continents soccer is part of a nation's culture resulting in large talent pools and huge financial resources, on the other continents soccer and especially talent identification and talent development programs are still in a developmental phase.

The performance of youth national teams is often seen as a key indicator of likely future international success at a senior level (3). However, especially in team sports, a substantial number of international elite athletes had no international experience in their youth careers (16). Similar results were found for soccer. Between a third and a half of soccer players with youth international experience in Germany became senior professionals suggesting that performance in youth is a limited indicator of senior success (9, 17).

It is interesting to note that academy U-team participation appears to be more important in the top-ranked soccer nations (3). There, high-quality soccer development programs are offered to those selected earlier from a young age, which gives them an advantage over those who are not selected or are selected later. Still, it remains unclear whether the most promising players are selected in these nations or whether those who are selected possessed the characteristics required to be selected at that time (attraction advantages) (12).

Remarkably, playing position has rarely been used as a dependent variable in sport talent studies. Herrebrøden and Bjørndal (3) found small effect sizes between U-team predictors and positions. Playing on U-19 teams did not predict future super-elite status for goalkeepers, which contrasts with the field players from top-ranked nations. Although many well-known FIFA World Players of the Year are forwards (e.g., Ronaldo, Messi), it has not been shown that forwards are more likely to play on national youth teams at younger ages than midfielders, defenders, or goalkeepers.

Of the eight quarter finalists at World Cup Qatar 2022, seven (i.e., France, Italy, Croatia, the Netherlands, Argentina, Brazil, Portugal) were established soccer nations. These nations have large talent pools, high soccer participation rates, many financial and logistical resources, and relatively high-level national competition (18). In all these nations, professional soccer academies select players under the age of 12. The selection of players at young ages in established nations might also be a result of the economic value of elite soccer. The UEFA Training Facility and Youth Investment Landscape report, published in 2020, revealed that youth soccer academies in Europe spend around €870 million per year (19). Globalization and increasing competition between youth soccer academies, who all fear missing out on the next superstar, also influence early selection. Each player in a soccer organization represents a value. Although only a few of the early players become successful, it appears to remain financially attractive to continue fielding teams of young players and selecting players under the age of 12.

We can conclude that much is still unknown about the age at which players representing their national team at the World Cup were selected to play in their youth for academies of professional soccer organizations. Therefore, the main aim of this study is investigate the ages at which national team players participating in World Cup Qatar 2022 started playing for a professional soccer academy and whether their starting age relates to continent and their current playing position (i.e., goalkeepers, defenders, midfielders, and forwards). It is hypothesized that most players in established soccer nations are selected from 6 to12 years of age while players raised in emerging soccer nations are mainly selected from 12 to 15 years of age. We also hypothesize that goalkeepers are selected at later ages than field players, regardless of the continent.

## Materials & methods

### Procedures

We conducted systematic desk research on all 32 teams who participated in FIFA World Cup Qatar 2022 and the individual players selected by those teams. Participating teams were allowed to select a maximum of 26 players for the tournament, which all but two nations actually did (France and Senegal selected 25 players).

We then conducted an online search to collect the following information about each player: name, current team, playing position at the World Cup, date of birth (day, month, and year), quarter in which they were born, the age and club at which the player first started playing professional youth soccer, and the organization for which they played. We used FIFA's Professional Soccer Report 2019 to determine whether an organization could be classified as professional, i.e., professional soccer academy. In most established and/or large nations, the first three leagues are generally classified as professional; in emerging and/or smaller nations, the first two leagues are generally classified as professional. In some nations (e.g., France, USA, South Korea) soccer organizations funded by the government, national soccer associations, clubs, or private donors [e.g., (former) professional players] are important institutions for talent development. When players from those nations were selected for soccer organizations, we used their age at selection.

The following search methods were used to determine players' starting ages. First, we searched Wikipedia.org (English version) for each player's name and information about their youth career and entry into a professional youth academy. If we found no or incomplete information, the search was extended to the Wikipedia page in the country's language (e.g., German Wikipedia for players from Germany). If we still did not find the information, we searched in Google for the player's name and added “youth career”. The sites most used to find more information were transfermarkt.com and soccerway.com. The validity of the data on transfermarkt.com was demonstrated before (20), and these sites are recently used more often to study professional football players (e.g., (21, 22). We also found information on biography sites and articles in national or local newspapers. To ensure correct analysis, all players were checked independently by two researchers. Only when both researchers found similar data for a player, the information found was classified as valid and reliable.

### Player careers and age effects

We looked at data about the start of the professional soccer careers of all the selected players (*N* = 829). Data was collected about the start of the professional career and starting age, defined as the year a player started playing for a youth team of a professional soccer club or a (academic) soccer academy. The players’ starting ages were assigned to one of the three age categories under 12, between 12 and 15 years old or older than 15. These age categories are primarily based on Côté (10) and Côté and Vierimaa (23) but slightly adjusted for the sampling and specializing years. The reason for adjusting the ages is that in most nations children start playing 11 against 11 from the under thirteen.

### Statistical analysis

The data was entered into Microsoft Excel 2016©. The median and interquartile ranges were expressed for the starting age at professional soccer academies and the current age at the World Cup. The variables were compared with the players' positions and the continent the national team represented based on the FIFA Qualifications, as well as the continent on which players started their career in a professional organization. Nonparametric tests were used since data were not distributed normally. A Kruskal–Wallis test was used to examine group effects for the age variables. In addition, pairwise (*post-hoc*) tests (Mann–Whitney U) were used to compare the FIFA continents on selection age. Chi-square tests were used to analyze whether the distribution of players starting at a professional academy during the sampling, specializing, or investment stage were related to continent or playing position. The level of statistical significance was set at *p *< 0.05 for all tests.

## Results

### Number of players included

In total, we found reliable information about the starting ages of 815 players in World Cup Qatar 2022. For 14 players, we had doubts about the information we found, or we could not find reliable and/or detailed information. All the researchers discussed what we had found and decided what to do with that information (i.e., use the age we found or note “no reliable information available”). Ultimately, we excluded these 14 players from our analysis.The 14 excluded players come from countries in Asia (*n* = 3), Africa (*n* = 10), and South America (*n* = 1). See Table 1 for the total number of players included per nation.

**Table 1:** Age and number of players in each phase at which players started playing for professional soccer academies, per continent and country.

Continent	Country	Median age [min-max]	*N* (missing)	Sampling (*n*)	Specializing (*n*)	Investment (*n*)
Africa	Total	**14.6 [4.2–21.8]**	**120 (10)**	**30 (24%)**	**45 (38%)**	**45 (38%)**
	Cameroon	17.2 [11.3–19.8]	26	1	10	15
	Ghana	14.8 [6.7–19.4]	26	5	12	9
	Morocco	13.9 [5.2–21.6]	26	11	6	9
	Senegal	13.3 [8.6–21.8]	20 (6)	6	10	4
	Tunisia	13.7 [4.2–21.8]	22 (4)	7	7	8
Asia	Total	**15.6 [7.0–21.3]**	**152 (3)**	**24 (16%)**	**65 (43%)**	**63 (41%)**
	Australia	15.2 [10.3–18.8]	26	3	13	10
	Iran	13.8 [10.0–20.3]	25	6	12	7
	Japan	15.8 [7.2–19.3]	26	5	11	10
	Qatar	16.9 [7.0–19.6]	26	7	4	15
	Saudi Arabia	15.9 [10.2–21.3]	23 (3)	2	11	10
	South Korea	15.7 [8.1–18.8]	26	1	14	11
Europe^a^	Total	**11.1 [4.2–22.6]**	**336**	**196 (58%)**	**108 (32%)**	**32 (10%)**
	Belgium	10.4 [5.2–15.6]	26	20	6	0
	Croatia	11.5 [6.2–17.1]	26	14	11	1
	Denmark	14.0 [8.3–17.2]	26	3	20	3
	England	8.4 [5.8–17.5]	25	21	2	2
	France	12.9 [6.3–16.7]	25	12	11	2
	Germany	11.0 [4.3–15.7]	26	16	10	0
	Poland	15.0 [6.4–21.1]	26	2	14	10
	Portugal	9.5 [6.8–20.0]	26	19	5	2
	Serbia	12.8 [4.8–18.9]	26	12	12	2
	Spain	9.4 [5.6–16.9]	26	20	4	2
	Switzerland	11.4 [7.3–16.5]	26	16	9	1
	The Netherlands	9.5 [6.2–19.8]	26	22	0	4
	Wales	8.9 [7.3–22.6]	26	19	4	3
North America and Central America	Total	**14.4 [4.2–19.5]**	**103 (1)**	**29 (28%)**	**39 (38%**	**35 (34%)**
	Canada	16.2 [9.9–19.2]	26	4	8	14
	Costa Rica	13.0 [8.3–16.8]	25 (1)	7	12	6
	Mexico	15.5 [6.8–19.5]	26	5	11	10
	USA	12.0 [4.2–17.8]	26	13	8	5
South America	Total	**12.9 [4.5–18.9]**	**104**	**37 (36%)**	**41 (39%)**	**26 (29%)**
	Argentina	11.8 [ 4.5–17.0]	26	13	8	5
	Brazil	12.8 [4.8–17.3]	26	10	10	6
	Ecuador	12.9 [7.2–18.7]	26	8	12	6
	Uruguay	14.0 [9.6–8.9]	26	6	11	9

^a^ The Kruskal–Wallis test revealed differences between continents on the starting age: *χ*^2^ (4) = 142.0, *p *< 0.001. *Post hoc* test showed that European players started playing earlier in professional soccer academies than players on all other continents (*p *< 0.05). Players from South America started earlier than players in Africa and Asia (*p *< 0.05). No differences in starting age were found between players from Africa, Asia, North America and Central America (*p *> 0.05).

### Starting age in a professional youth soccer organization

Players participating in World Cup Qatar 2022 started playing for a soccer academy at a median age of 13.2 years (range: 4.2–22.6 years). The lowest median age was found in England (8.4), while the oldest median age was found in Cameroon (17.2). On most continents, no player started before the age of 4; in Asia, the youngest age was 7. The oldest player to start playing for a professional organization was 22 years old. Regardless of continent, nine players participating in the World Cup Qatar 2022 teams were 18 years of age or older starting to play for a professional soccer organization (i.e., these players were in their youth not selected for a professional soccer academy) Table 1 presents the median age of players per nation and continent.

### Starting age and continent

**Figure 1:**
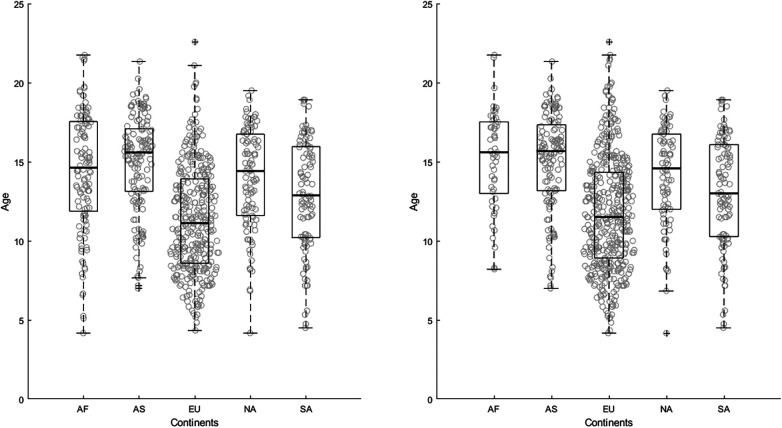
The left panel (**A**) shows data about the ages at which players started their career in a professional soccer academy. The continent designation was based on the location of the country they played for at the World Cup. The right panel (**B**) shows data about the ages at which players started their career in a professional academy, continent was based on the country where they started this career. Abbreviations for continents: AF, Africa; AS, Asia; EU, Europe; NA, North America and Central America; SA; South America.

The Kruskal–Wallis test revealed differences between continents on the starting age: *χ*2 (4) = 142.0, *p *< 0.001. The *post hoc* test showed that European players [11.1(4.2–22.6)] started playing for professional soccer academies at younger ages than players on other continents (*p *< 0.05). Besides Europe, the players from South America [12.9(4.5–18.9)] started earlier than players from nations in Africa [14.6(4.2–21.8)] and Asia [15.6(7.0–21.3)] but not earlier than players from North America and Central America nations [14.4(4.2–19.5)]. We found no differences in starting age between players from Africa, Asia, North America and Central America (*p *> 0.05). See also Figure 1A.

During the analysis, it became clear that 145 players did not start playing soccer for a professional academy in the country they represented at the World Cup. Most of these players were born and/or raised in the country of their soccer educational program since their (grand)parents immigrated here, and as of such have dual nationality, Furthermore, of those players, 104 had started playing for an organization on another continent (mainly Europe). This was most prominent for Africa: 70 players (56%) started on another continent, mainly in Europe [56% (*n* = 67)]. We performed a Mann–Whitney *U*-test to compare the median age of African players who started playing in Africa [15.6(8.3–21.8)] to those who started in Europe [13.6(4.2–21.8)]. The test revealed no statistical difference between starting ages (*p *= 0.092). See also Figure 1B.

**Figure 2:**
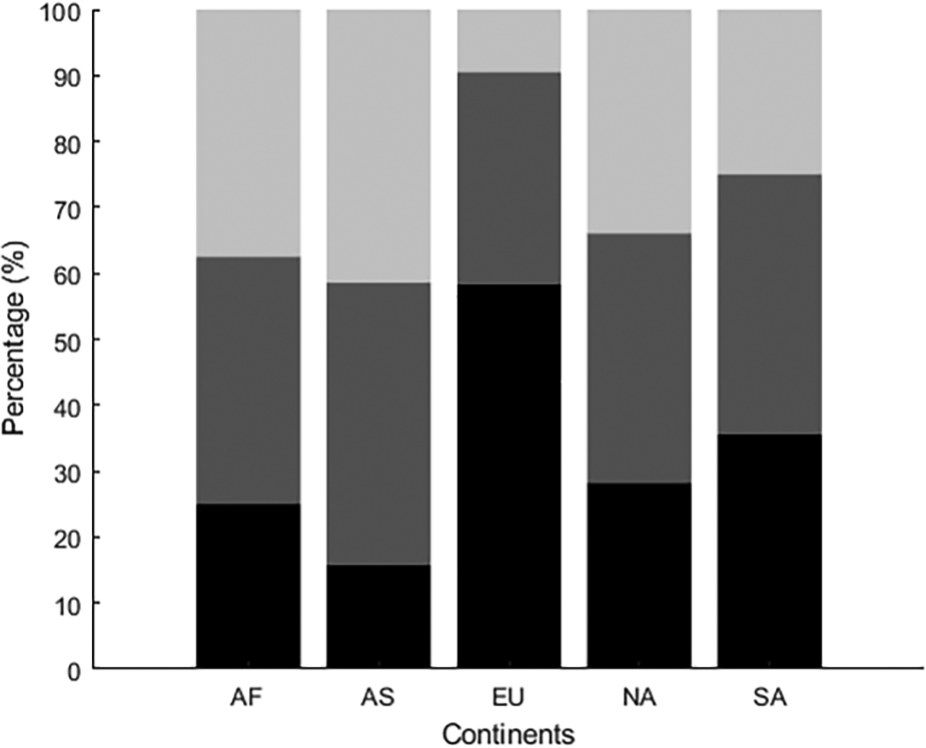
The relative distribution of players per continent who started their career at a professional soccer academy in the sampling phase (black), specializing phase (dark gray), or investment phase (light gray). Abbreviations for continents: AF, Africa; AS, Asia; EU, Europe; NA, North America and Central America; SA, South America.

The proportion of players that started in the different stages (sampling, specializing, or investment) differed between continents [*χ*^2^ (8) = 126.9, *p* < 0.001, see Figure 2]. In Europe, 58% of all players (*n* = 196) started playing soccer for a professional soccer academy in the sampling stage. Still, 10% (*n* = 32) of the European players started over the age of 15, and about a third (*n* = 108, 32%) were between 12 and 15. In South America, most players (*n* = 41, 39%) started in the specializing years (between ages 12 and 15). The next largest group began in the sampling years (*n* = 37, 36%) and then the investment years (*n* = 26, 25%). In both Asia and North America and Central America, most players entered the youth professional academies between the ages of 12 and 15 (*n* = 65, 43% and *n* = 39, 38%, respectively), while the fewest number of players started at the sampling stage. In Africa, 45 players (38%) started in the specializing years and an equal number started in the investment years. 25% of players from African nations (*n* = 30) started playing before age 12 (see Table 1).

### Starting age and player position

**Figure 3:**
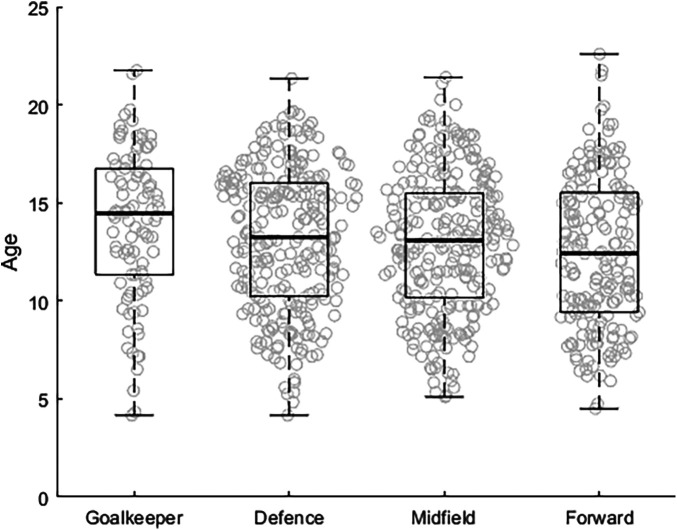
Median age at which players started their career in a professional soccer academy in relation to their position.

**Figure 4:**
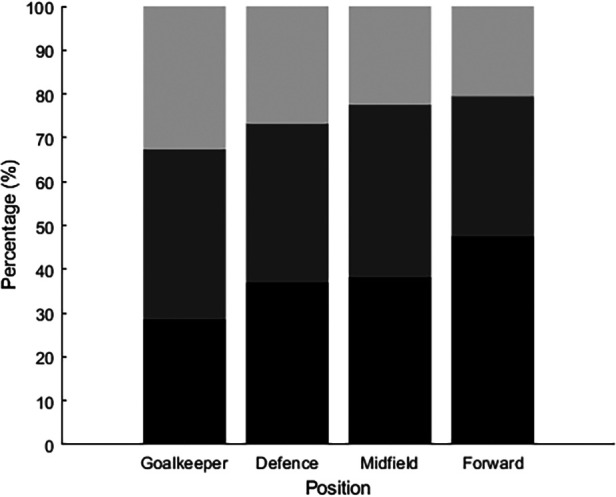
The relative distribution of players per playing position who started their career at a professional soccer academy in the sampling phase (black), specializing phase (dark gray) or investment phase (light gray).

Most players participating in World Cup Qatar 2022 were labeled midfielders (*n* = 267), followed by defenders (*n* = 265), forwards (*n* = 185), and goalkeepers (*n* = 98). The Kruskal–Wallis test revealed differences between players' playing position at their starting age: *χ*^2^ (3) = 11.7, *p *< 0.05. The *post hoc* test showed that forwards [12.4(4.5–22.6)] started playing at younger ages than goalkeepers [14.5(4.2–21.8)]. We found no differences between midfielders [13.1(5.2–21.4)], defenders [13.3(4.2–21.3)], and goalkeepers in terms of their starting age (see Figure 3). In addition, the proportion of players that started in the three stages (sampling, specializing, or investment) differed between position (*χ*^2^ (6) = 13.2, *p* = 0.040 (see Figure 4); forwards were more likely to start playing in the sampling stage.

## Discussion

The main aim of this study was to discover the ages at which national team players participating in World Cup Qatar 2022 started playing for a professional soccer academy and whether their starting age relates to continent and their current playing position (i.e., goalkeepers, defenders, midfielders, and forwards). For most players (*n* = 815) we found reliable data about the age they started playing for a professional soccer academy. This study revealed that:

1. World Cup players start playing for a professional soccer academy at a median age of 13.2 years.
2. The difference between the earliest (4) and latest starting age (20) is 18 years of age. Ths differences in starting age and years in an organization reveal the different pathways to the highest level in soccer.
3. There are differences between continents in starting age. In Europe, where soccer is generally the most popular sport and millions of youngsters play for excellent leagues and clubs, most national team players entered youth academies in the sampling years (before age 12). On the other continents, most players entered organizations between the ages of 12 and 15 (i.e., the specializing years).
4. A significant number of players from African nations were born and raised in Europe and/or learned to play soccer in Europe. For example, of the 26 players for Morocco—which placed 4th in the World Cup—15 were born and raised in Europe.
5. Age of entrance in a professional talent development program is not related to the playing position of elite players. We only found a small difference between forwards and goalkeepers.

As far we know, this is the first study to look at the age at which all the players in a FIFA World Cup tournament began playing for a professional soccer academy. The results add to studies (e.g. (2, 8), that focus on elite athletes’ junior careers and sport success with a specific focus on starting age. In line with our hypothesis and Hornig et al. (8), players in more established soccer nations start playing for professional youth organizations in the sampling years, before age 12.

For example, look at the 2022 World Cup winner, Argentina. Besides Lionel Messi (who began playing in a professional soccer academy at age 7), 12 other players for Argentina started playing in the sampling years. However, five players for Argentina started after the age of 15, which supports Wenger's statement that some players might surprise in later phases (24). We found similar results for Argentina's opponent in the World Cup final, France. 12 players started before the age of 12, but a significant number of players started later.

Argentina and France seem to be typical for established soccer nations. While most players in those countries enter professional soccer academies at young ages, a significant number of players begin later. Although our results could be interpreted to suggest that it is important to start playing for a professional soccer academy at an early age, it should also be acknowledged that entering youth soccer academies at a young age (<12) is not a prerequisite for eventual performance at the highest level in soccer. The differences in starting age also emphasize the need for policy makers and sport managers to adopt a balanced approach to selection-based systems and unstructured grass roots (soccer) activities. Players’ development is a non-linear trajectory, and a too strict selection-based system will result also in the deselection of many players with the potential to excel in the future too. Still, many questions remain, and it is recommended, supported by FIFA, to conduct a worldwide organizational and cultural study of current talent identification and talent development systems in soccer.

Our finding that players from established soccer nations are likely to get an early start at professional soccer academies confirms the findings of a study about the German national team players (8). In European nations like the Netherlands, Germany, Spain, and England, 62%–85% of players on the national team first started playing for a professional soccer organization during the sampling years. Early selection frequently results in receiving more and better opportunities to develop because a player's teammates are better, they have better coaches, better opponents, and better training facilities, and they engage in more deliberate practice. However, we have no information about the content of the programs offered to children in the youngest age groups. Beginning around ages 10–12, several professional soccer academies implement a diverse program (e.g., practicing other sports like judo and gymnastics, playing soccer on different surfaces), and an early start does not have to result in early specialization (13, 25). Whether early entrance results in the best development of youngsters remains an open question and warrants more research.

Although players from the most successful nations at the World Cup start young, emerging nations should not necessarily decide to copy more established nations' systems. Differences in the number of talented players, the accessibility of training facilities, and the availability of financial and logistical resources create different circumstances which might require a different approach (18). Also, we should be aware that our descriptive and retrospective design makes our study susceptible to the survivorship bias, i.e., a form of selection bias which occurs when focusing on a specific selected group of people. The players who made it to the World Cup are, at least partly, the result of chosen talent identification and talent development systems. Whether this is “the best system” could not be answered based on the results of our study.

When we examined the relationship between age and playing position, we only found a difference between the median age at which forwards and goalkeepers first started playing for a professional soccer academy, and that difference appears to be small. Playing position has not been included as a variable in other talent identification studies, except for Herrebrøden and Bjørndal (3). They suggest a plausible explanation for the slightly later age at which goalkeepers join professional youth soccer: initially, goalkeepers are strongly rewarded by growth and maturation, and at later ages they are rewarded for their skills. Our study found that several goalkeepers started playing at professional soccer academies at young ages (e.g., German goalkeepers Manuel Neuer and Marc-André ter Stegen began at age 5). However, we do not know whether they were selected for the professional organization as a keeper or as a field player.

Several reasons could explain the lack of differences in the starting ages of forwards, midfielders, and defenders. First, we only have information about the players' playing position at the World Cup; we do not know what position they played in their youth. Soccer is a team sport and players are initially selected at the same age to join a youth team. It is possible that many young players are identified based on their generic soccer skills (e.g., technical skills, ball handling skills, understanding of the game, physical skills) and only later begin to specialize in a specific position, based on their later development. Also, in many Dutch professional soccer academies, youth players play at different lines and positions until U16, and players' specific qualities only emerge later. Second, performing at the youth level requires different qualities than performing at the elite level (1). It is reasonable to expect that players who will successfully compete at the elite level will only exhibit the specific skills that fuel that success (e.g., attacking, defending, turnovers) at later ages. Third, there is evidence that skills like perseverance, commitment, and self-regulation help to determine the eventual success (or not) of talented youth players (26–28). These skills are not directly related to playing position. However, research into these skills is scarce (29), and future studies on talent identification and talent development should include playing position to get a better understanding.

In line with our hypothesis, we found differences between continents. On average, players in European nations start playing for professional soccer academies at younger ages than players on other continents. European nations not only start selecting players in organized soccer from young ages, but their large talent pools, good financial and logistical resources, well-developed sport infrastructure, and good coaching education programs could explain the early start of youngsters in professional youth organizations (4, 18). However, there are also differences within Europe: Denmark and Poland are outliers compared to most other European nations with players started later. Denmark, as other Scandinavian countries, has a voluntary broad-based sports model which might explain why players start playing for professional soccer academies at later ages. In Poland many soccer players first start training in commercial youth soccer academies. Professional soccer clubs' academies select players most often from those commercial academies. In Asia, North America and Central America, many nations have a school-sport system which starts at later ages and could explain the differences we found. In Africa, there is still a lack of well-developed sport structures and no or weakly organized talent identification and talent development programs. Therefore, it is unsurprising that more than half (*n* = 70) of the national team players representing African nations were raised and/or started playing for a professional youth soccer organization in a country other than the one for which they played in World Cup Qatar 2022. Given FIFA's ambition to develop soccer globally, it would be of interesting to discover whether more structured systems can be implemented and whether those would result in more organized talent identification and talent development systems and finally success in international competitions.

A main strength of our study is our focus on players from all nations that participated in World Cup Qatar 2022. A lot of information about elite soccer players is available on the internet, including the first steps in their soccer careers. However, this is also the main limitation of our study. Our main source of player data was Wikipedia, an online encyclopedia whose content is written by volunteer authors. Although information on Wikipedia can be controlled and the site requires the use of reliable sources, some information about players' starting ages may be incorrect. When we had doubts about the information given for a player, we searched for more information on other websites and discussed the findings with all researchers. It was sometimes difficult to find reliable information about players' starting age from emerging nations, so we did not include data about 14 players. A second limitation inherent to the retrospective approach is that we could not calculate the chance of becoming an elite soccer player as a function of selection age. Also, we did not study whether players were deselected after their first selection and how their pathways emerged. Güllich (2014) showed that most professional soccer players in Germany are chosen through a collectivistic approach (i.e., repeated procedures of selection and deselection through childhood and youth) (9). Although determining this relationship was not the aim of this study, more information about World Cup players' pathways would further help us understand talent identification and talent development processes.

To summarize, this study –which included almost all World Cup Qatar 2022 players—showed that the players started playing for professional soccer academies at a median age of 13.2. We noticed differences in starting age between continents, but not between playing positions. European players (11.1 years) started playing for professional soccer academies at younger ages than players on other continents. Players from South America (12.9), especially Argentinian and Brazilian players, started earlier than players form nations in Africa (14.6) and Asia (15.6). We also found an extremely large range of ages—between 4 and 22—at which players started, regardless of playing position and continent, indicating the different pathways to the highest level in soccer. Several studies have criticized the early selection of soccer players. However, our study shows that most World Cup Qatar 2022 players from established soccer nations started playing for a professional soccer academy in their sampling years (between ages 6 and 13 years of age). Whether this early start in established nations is required to become an elite soccer player or whether the current system results in an early start remains an open question. At least, our findings should reassure players from all soccer nations that an early start in a professional soccer academy is not a prerequisite for participation in FIFA's World Cup.

## Data Availability

The raw data supporting the conclusions of this article will be made available by the authors, without undue reservation.
